# Effects of Cobra Cardiotoxins on Intracellular Calcium and the Contracture of Rat Cardiomyocytes Depend on Their Structural Types

**DOI:** 10.3390/ijms24119259

**Published:** 2023-05-25

**Authors:** Alexey S. Averin, Alexey V. Berezhnov, Oleg Y. Pimenov, Miliausha H. Galimova, Vladislav G. Starkov, Victor I. Tsetlin, Yuri N. Utkin

**Affiliations:** 1 Institute of Cell Biophysics, Federal Research Center “Pushchino Scientific Center of Biological Research”, Pushchino Branch, Russian Academy of Sciences, Pushchino 142290, Russia; averinas82@gmail.com (A.S.A.); alexbereg56@gmail.com (A.V.B.); 2 Institute of Theoretical and Experimental Biophysics, Russian Academy of Sciences, Pushchino 142290, Russia; polegiteb@gmail.com (O.Y.P.); mgalimova@mail.ru (M.H.G.); 3 Shemyakin–Ovchinnikov Institute of Bioorganic Chemistry, Russian Academy of Sciences, Moscow 117997, Russia; vladislavstarkov@mail.ru (V.G.S.); victortsetlin3f@gmail.com (V.I.T.)

**Keywords:** cardiotoxin, cardiomyocyte, hypercontracture, calcium concentration

## Abstract

Cardiotoxins (CaTx) of the three-finger toxin family are one of the main components of cobra venoms. Depending on the structure of the N-terminal or the central polypeptide loop, they are classified into either group I and II or P- and S-types, respectively, and toxins of different groups or types interact with lipid membranes variably. While their main target in the organism is the cardiovascular system, there is no data on the effects of CaTxs from different groups or types on cardiomyocytes. To evaluate these effects, a fluorescence measurement of intracellular Ca^2+^ concentration and an assessment of the rat cardiomyocytes’ shape were used. The obtained results showed that CaTxs of group I containing two adjacent proline residues in the N-terminal loop were less toxic to cardiomyocytes than group II toxins and that CaTxs of S-type were less active than P-type ones. The highest activity was observed for *Naja oxiana* cobra cardiotoxin 2, which is of P-type and belongs to group II. For the first time, the effects of CaTxs of different groups and types on the cardiomyocytes were studied, and the data obtained showed that the CaTx toxicity to cardiomyocytes depends on the structures both of the N-terminal and central polypeptide loops.

## 1. Introduction

**Figure 1:**
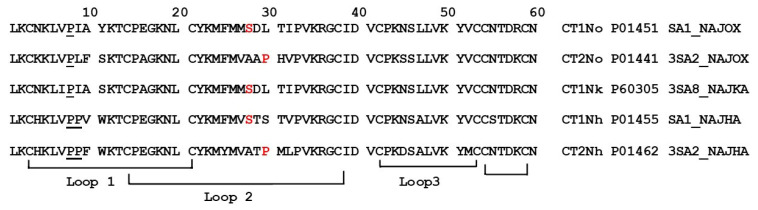
Amino acid sequences of CaTx used in this work. CT1No and CT2No are the cytotoxin 1 and cytotoxin 2, respectively, from *Naja oxiana* cobra venom. CT1Nk—cytotoxin 1 from *N. kaouthia* cobra venom. CT1Nh and CT2Nh are cytotoxin 1 and cytotoxin 2, respectively, from *N. haje* cobra venom. Serine 28 and proline 30 are shown in red, proline residues in N-terminal loop are underlined.

Cobra venom cardiotoxins (cytotoxins, CaTx) are small proteins belonging to the family of three-finger toxins [1]. They are one of the main components of cobra venoms and, in the victim organism, affect the cardiovascular system. The structure of the CaTx molecule is characterized by three β-structured polypeptide loops emerging from a small hydrophobic core stabilized by four disulfide bonds. According to differences in the interaction with zwitterionic phospholipid dispersion, S- and P-types of CaTx were distinguished [2]. The toxins of the S-type comprise a serine residue at position 28 (Figure 1) of the amino acid sequences, and never have proline at position 30, at which usually leucine, lysine, or serine residues are located. CaTXs of the P-type contain a proline residue at position 30 and alanine at position 28 in most sequences. On the basis of structural studies, CaTx were classified in two other subclasses, i.e., groups I and II [3,4]. Group I CaTx is characterized by the presence of two proline residues at position 8 and 9 as well as an aromatic residue (tyrosine or tryptophan) at position 11 of the amino acid sequence. All other CaTxs were of group II.

The toxins from different groups in both classifications are distinguished by different biological activities. The available data show that toxins of both P- and S-types disturb the lipid bilayer of anionic-phospholipid-containing membranes, but with different efficiency; P-type toxins damage the lipid bilayer more strongly [5]. If we consider groups I and II, then the toxins of group II have a greater membrane-disrupting activity [6].

At the level of whole organisms, one of the main targets of CaTxs is the cardiovascular system, both the heart and blood vessels being affected by toxins [7,8,9]. There are only limited data on the direct effects of CaTxs on the function of cardiomyocytes. Thus, using fluorescent indicators, it was shown that 1 µM CaTx caused a significant increase in the concentration of Ca^2+^ ions in the cytosol [10,11]. This increase was accompanied by a disturbance of the contractions and shape of cardiomyocytes and, as a result, led to contracture [10,11,12]. We have previously shown that the P- and S-types of CaTx have different effects on rat papillary muscle and aorta [8], as well as on the rat heart [7]. It was found that cardiotoxin 2 (CT2No) from cobra *Naja oxiana*, CaTx of the P-type, is noticeably more active than cardiotoxin 1 (CT1No), CaTx of the S-type from the same cobra. Since there were no data on the effect of different types and groups of CaTxs on cardiomyocytes, we compared the effects of several CaTxs on rat cardiomyocytes.

## 2. Results

Since the cardiotoxic effects of CaTxs are associated with intracellular Ca^2+^ dysregulation and with Ca^2+^ overload of the cells [10], we used a fluorescence measurement of intracellular Ca^2+^ concentration and an estimation of the cell shape change to assess the CaTx effects. In this work, a comparative study of the effect of five CaTxs on cardiomyocytes of the rat left ventricle was carried out. CaTxs were isolated from the venoms of three cobra species. Their amino acid sequences are shown in Figure 1. CT1No, CT1Nk, and CT1Nh belong to S-type, and CT2No and CT2Nh—to P-type. CT1Nh and CT2Nh are from group I, all others—from group II. 

**Figure 2:**
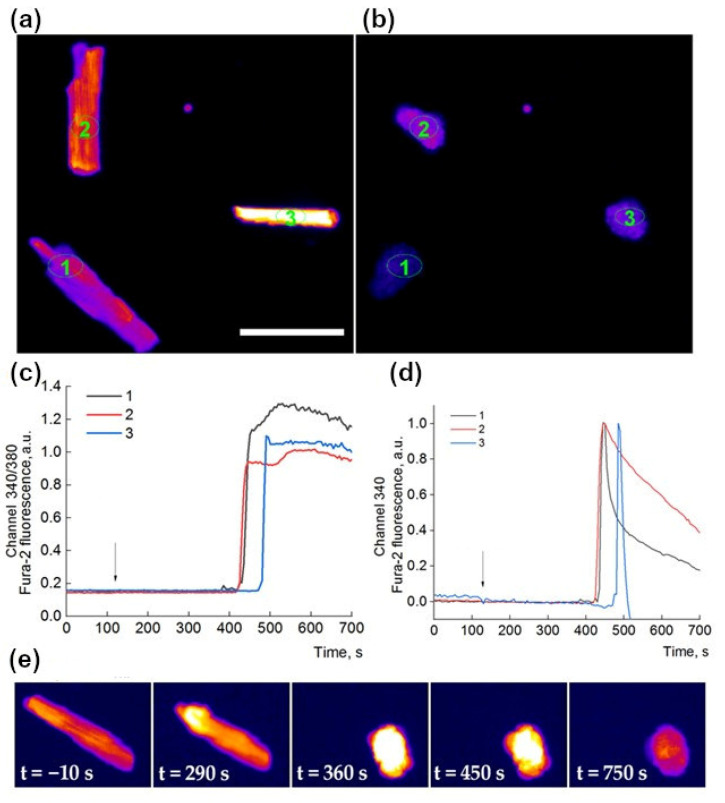
Examples of the development of cardiomyocyte Ca^2+^ overload, accompanied by HC, under the influence of cardiotoxin. Representative images of three normal cardiomyocytes loaded with Fura-2 (channel 380 only, LUT Fire) (**a**) and the same hypercontracted cardiomyocytes 380 s after the application of 25 µM CT2No (**b**). Numbers 1–3 denote individual cardiomyocytes. Ovals depict regions of interest (ROIs). Intracellular ROIs are used for monitoring [Ca^2+^]i responses and are selected manually. Scale bar—100 µm. The increment in [Ca^2+^]i registered as 340/380 ratio (**c**) and in 340 channel (**d**). Arrows indicate application of 25 µM CT2No. (**e**) Development of the effect over time after application of 25 µM CT1Nk at t = 0.

We have previously found that CT1No and CT2No, being of S- and P-type, respectively, have adverse effects on the rat papillary muscle and heart [7,8], CT2No manifesting higher toxicity. To find if this difference exists at the cellular level, the effects of these and three other CaTxs on the rat cardiomyocytes were studied in the present work. For this purpose, the changes in the concentration of intracellular Ca^2+^ and cell shape were investigated. In the course of preliminary experiments when studying CaTxs effects on isolated rat cardiomyocytes, we found that, at a concentration of 25 µg/mL, both toxins produced a cytotoxic effect. This concentration was then used for the initial comparison of toxin activities. To determine the intracellular Ca^2+^ dynamics, a ratiometric fluorescent dye Fura-2, which binds to free intracellular calcium, was used. Several isolated cardiomyocytes were monitored individually under the microscope. The results of the typical experiment are shown in Figure 2. As can be seen in Figure 2, in normal cardiomyocytes (Figure 2a), a while after the addition of CaTx, the level of intracellular Ca^2+^ begins to increase rapidly (Figure 2c,d) and, at the same time, the cardiomyocyte begins to contract. The calcium overload results in the development of irreversible hypercontracture (HC), which was observed previously under the action of different toxic agents [13,14] and subsequent dye loss; while, normally, the contraction of a cardiomyocyte usually does not exceed 10% of its length, HC is characterized by a sharp, 2–3 times decrease in the length of the cardiomyocyte and the transition of its shape to an almost spherical one (Figure 2b,e) [15,16]. The cell loses its characteristic structure and the level of fluorescence begins to decrease due to the release of the dye from the cell (Figure 2e).

**Figure 3:**
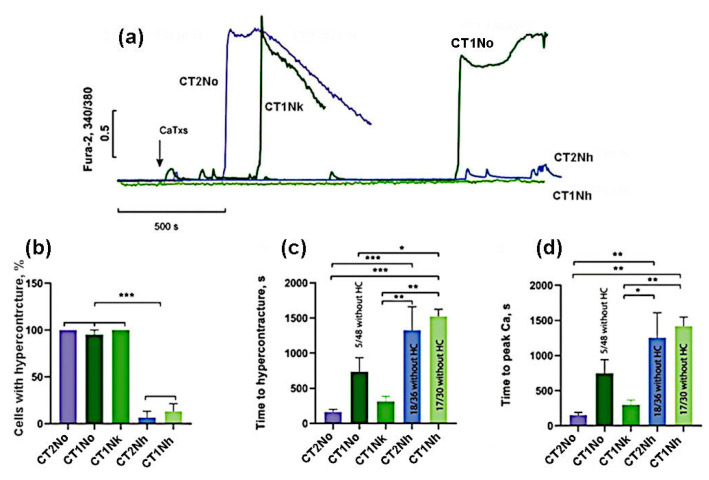
Comparison of the effects of CaTxs at a concentration of 25 µg/mL. (**a**) Typical example of recording a fluorescent Fura-2 signal. Arrow indicates cardiotoxin application. (**b**) Number of cells with hypercontracture (HC) after 30 min of CaTx application. (**c**) Time of the onset of the HC. (**d**) Time to the maximum rate of increase in concentration of intracellular Ca^2+^. CT2No (*n* = 4); CT1Nk (*n* = 4); CT1No (*n* = 4); CT2Nh (*n* = 3); and CT1Nh (*n* = 3). Asterisks indicate the significant differences at * *p* < 0.05, ** *p* < 0.01, and *** *p* < 0.005.

At a concentration of 25 μg/mL, CT2No and CT1Nk induced HC in 100% of cells while, for CT1No, the proportion of cells with HC was 89 ± 13% (Figure 3). CT1Nh and CT2Nh showed a significantly weaker activity, causing HC in 18 ± 17% and 7 ± 12% of cells, respectively (Figure 3b). It should also be noted that the time of HC onset and the rise in intracellular Ca^2+^ concentration was the shortest for CT2No, being 162 ± 86 and 152 ± 87 s. The same parameters for other toxins were: 312 ± 148 and 299 ± 137 s for CT1Nk; 736 ± 397 and 747 ± 387 for CT1No; 1328 ± 577 and 1255 ± 615 for CT2Nh; and 1522 ± 177 and 1420 ± 223 s for CT1Nh. Thus, the toxins studied were divided into 2 groups:

(1) the group with high activity, which included CT2No, CT1Nk, and CT1No;
(2) the group with low activity: CT2Nh and CT1Nh.

**Figure 4:**
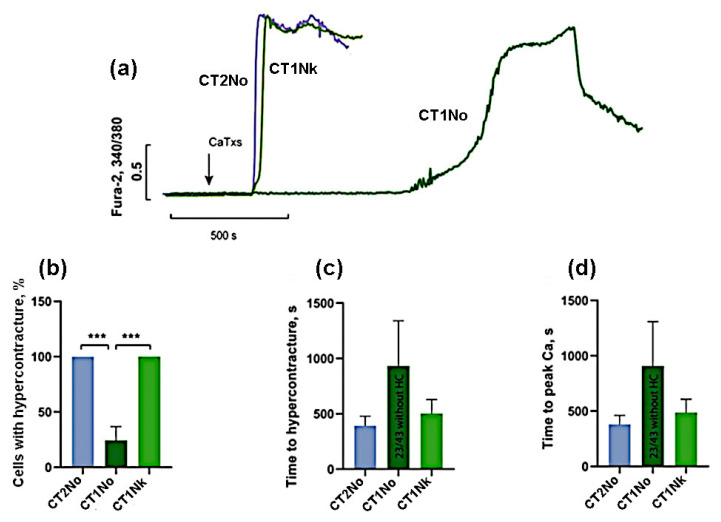
Comparison of the CaTx effects at a concentration of 10 µg/mL. (**a**) Typical example of recording a fluorescent Fura-2 signal. An arrow indicates application of cardiotoxins. (**b**) Number of cells with HC after 30 min of CaTx application (**c**) Time of the onset of the HC. (**d**) Time to the maximum rate of increase in concentration of intracellular Ca^2+^. CT2No (*n* = 6); CT1Nk (*n* = 6); and CT1No (*n* = 3). Asterisks indicate the significant differences at *** *p* < 0.005.

For a more accurate comparison of the activities for the toxins of the first group, we carried out additional experiments at a lower (10 μg/mL) concentration of toxins. As can be seen from Figure 4, at this concentration, cardiotoxins CT2No and CT1Nk caused HC in 100% of cells while, for CT1No, the number of cells with HC was 24 ± 21%. The time of onset of HC and the rise in intracellular Ca^2+^ was the shortest for CT2No (393 ± 207 and 396 ± 217 s); it was slightly longer for CT1Nk—503 ± 307 and 520 ± 318 s, and reached 933 ± 707 and 909 ± 634 s for CT1No. These results suggest that CT2No and CT1Nk toxins are very similar in their ability to induce HC and Ca^2+^ overload in cardiomyocytes and are significantly more active than CT1No. 

**Figure 5:**
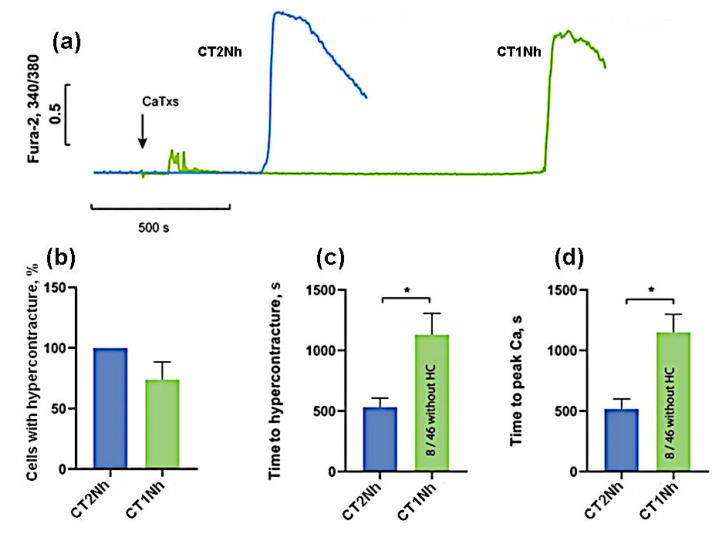
Comparison of the effects of CaTxs at a concentration of 100 µg/mL. (**a**) Typical example of recording a fluorescent Fura-2 signal. An arrow indicates the application of cardiotoxins. (**b**) Number of cells with HC after 30 min of CaTx application. (**c**) Time of the onset of the HC. (**d**) Time to the maximum rate of increase in the concentration of intracellular Ca^2+^. CT2Nh (*n* = 4) and CT1Nh (*n* = 4). Asterisks indicate the significant differences at * *p* < 0.05.

To compare CT2Nh and CT1Nh toxins possessing fewer pronounced cytotoxic properties, their higher concentrations (100 µg/mL) were used. As can be seen in Figure 5, at this concentration, CT2Nh induced HC in 100% of cells while, for CT1Nh, the proportion of cells with HC was 74 ± 29%, but this difference is not statistically significant. The time of onset of HC and the time of rise in intracellular Ca^2+^ for CT2Nh were 531 ± 148 and 518 ± 163 s, and for CT1Nh these times were 1132 ± 342 and 1151 ± 295 s, respectively, the observed difference between the two toxins being statistically significant. These results suggest that CT2Nh is much stronger than CT1Nh in its cytotoxic activity against cardiomyocytes.

The data obtained (summarized in Table 1) allow us to propose the following series of CaTx cytotoxicity against rat cardiomyocytes: CT2No=CT1Nk>CT1No>CT2Nh>CT1Nh.

## 3. Discussion

The homeostasis of Ca^2+^ ions plays an important role in the functioning of the heart in general and cardiomyocytes in particular. The disturbance of calcium homeostasis in cardiomyocytes can lead to the appearance of uncontrolled heart contractions and, thus, a disruption of the normal function of the heart muscle. The available evidence indicates that cobra CaTxs disrupt the calcium homeostasis of cardiomyocytes. The action of toxins results in a calcium overload followed by HC and cell death [10]. The action of cardiotoxins on the heart muscles leads to a disruption of their contractions and contracture and, by acting on the heart, these toxins cause a systolic heart arrest. Earlier, we studied the effects of CT1No and CT2No on the rat papillary muscle and showed that both toxins produced contracture at a concentration of 5 μg/mL [8]. These CaTxs are of different types, CT1No of S-type and CT2No of P-type, and, against papillary muscle, CT2No was more active. On the isolated rat heart, perfused according to the Langendorff technique, CT2No was also more active than CT1No [7]. In the present work, we extended our studies to cardiomyocytes and supplemented the set of toxins by three more CaTxs. 

As mentioned in the introduction, the CaTxs are the main components in the majority of cobra venoms. For example, in the venom of the Nigerian *N. nigricollis* cobra, CaTxs comprise more than 70% of proteins [17]. As concerns the toxins used in our study, the content of CT1No and CT2No in the crude *N. oxiana* venom is 15 and 18%, respectively [18]. According to the proteomic data, CT1Nk accounts for more than 20% of all proteins in the *N. kaouthia* venom [19]. The proteomic analysis of *N. haje* venom showed that the three-finger toxins to which CaTxs belong constitute 52% of the total venom proteins [20], and CaTxs make up more than 50% of the three-finger toxins [21]. That is, the content of CT1Nh and CT2Nh in the *N. haje* venom is a few dozen percent.

The toxin concentrations applied here for cardiomyocytes were higher than those (5 µg/mL) affecting the function of the papillary muscle and heart. This difference can be explained by the fact that cardiomyocytes were at rest, while the papillary muscle contracted rhythmically under the influence of electrical stimuli. Since, in work on mouse cardiomyocytes, an increase in the frequency of stimulation increased the diastolic calcium level and calcium content in the sarcoplasmic reticulum (SR) [16], it could be expected that, under conditions of electrical stimulation to trigger contracture, a lower influx of calcium induced by cardiotoxins would be required. All the toxins studied in this work produced an increase in intracellular calcium and the development of contracture, these effects being similar to those observed earlier [10]. The release of calcium from the SR and the entry of extracellular calcium through various mechanisms contribute to the creation of calcium overload [8,10]. Earlier, on the isolated rat heart, perfused according to the Langendorff technique [9], we observed that CT1No and CT2No produced a transient increase in pulse pressure and an increase in diastolic pressure without changing the heart rate. Finally, both toxins caused heart contracture. It may be expected that the effects of other CaTxs studied in the present work on the whole heart will be similar. 

The diversified actions of cardiotoxins should also be noted. In addition to calcium overload, they can interact with mitochondria, disrupting their function [22,23], which may also cause cardiotoxic effects [14]. However, as we have shown earlier [8], it is the blocking of the entry of extracellular calcium that prevents irreversible damage to the myocardium. That is, it is this process that plays a leading role in the development of pathological disorders. A further chain of pathological disorders may include the increase in reactive oxygen species [23], as well as the activation of peptidases [24,25], leading to irreversible contracture and cell death [10,26]. Cardiomyocyte HC is an excessive cell shortening. The development of cardiomyocyte HC is an essential mechanism of the reperfusion-induced injury. HC can propagate to adjacent cells through gap junctions [27] and is an important mechanism of myocyte death during reperfusion. Reperfusion-induced HC may originate from Ca^2+^ overload, when energy recovery is rapid but cytosolic Ca^2+^ load is high [28,29]. A similar phenomenon is seen in the action of CaTxs on cardiomyocytes. It should be mentioned that reperfused myocardial infarcts consist almost exclusively of areas of contraction band necrosis formed by hypercontracted dead cardiomyocytes [30]. Cobra CaTxs causes cardiomyocyte HC and death, and this situation is similar to that which one might see in reperfusion heart injury.

The available data suggest that the in vivo toxicity of CaTxs can vary quite a lot (e.g., [31]), but it may depend on many parameters, not only on the CaTx effects on the heart. It is even more difficult to interpret the changes that occur in the cardiovascular system when exposed to the whole venom since, in addition to cardiotoxins, cobra venom contains neurotoxins, PLA2, and many other components [32,33] which can affect the heart. In experiments on mice, the venom of the cobra *N. sputatrix* had a pronounced cardiotoxic effect, causing bradycardia, an increase in the amplitude of QRS complexes, and cardiac arrhythmias [34]. However, it is difficult to accurately determine this effect exclusively with cardiotoxins since cardiotoxic effects have been shown for many PLA2s [35,36].

In clinical practice, cobra bites tend to be dominated by neurotoxic effects, and the main efforts are directed towards maintaining respiration, with much less attention paid to cardiovascular effects [37,38]. Nevertheless, there are cases with pronounced cardiotoxic effects [39,40]. At the same time, as a result of therapy directed primarily against neurotoxic components, the significance of cardiotoxic effects increases [41]. This leads to the idea that the identification of the most active cardiotoxins can be useful in understanding the species specificity of the development of the pathological effects of cobra envenomation and can provide an additional criterion for evaluating the effectiveness of antivenoms.

The comparative data on the direct effects of various cardiotoxins on cardiac muscle or on the heart are very limited. As already noted, we showed that a P-type cardiotoxin had greater cardiotoxicity than that of the S-type [7,8]. This difference is explained by the greater membrane-damaging effect of the P-type CaTxs as compared to the S-type ones [5], i.e., the structure of the central polypeptide loop II (Figure 1) determines the CaTx activity. Recently, we demonstrated that the N-terminal loop I also plays an important role in the membrane-damaging activity of CaTxs [6]. We found that CaTxs of group I with a Pro–Pro peptide bond in loop I exhibited attenuated membrane-perturbing activity in model membranes, and lower cytotoxic/antibacterial activity than the CaTsx of group II with a single Pro-residue in the loop I [6]. In the present work, we show that differences in the structure of both loop I and loop II determine the cardiotoxic effects of CaTxs. Therefore, CT2No demonstrated the highest toxicity to cardiomyocytes, this toxin being of the P-type from group II. The least active was CT1Nh of the S-type from group I. Three other CaTxs occupied intermediate places depending on the type and group (Table 1). 

Interestingly, S-type CT1Nk was more potent than the S-type CT1No. The main differences between these two toxins are in the structure of loop I. Thus, Ser-11 in CT1Nk is changed to Tyr-11 in CT1No (Figure 1). Such a bulky aromatic residue may complicate the insertion of the loop into the membrane, reducing the general cytotoxic effect. The difference in the activity between CT1Nk and CT1No underlines once more the importance of the loop I structure for the activity of CaTxs. 

Although we found a variation in the effects of CaTxs belonging to different types and groups, still only a limited number of toxins of each type and group was used in this work. To find out whether the differences we discovered extend to other toxins, we plan to carry out a study on a larger number of CaTxs.

## 4. Materials and Methods

### 4.1. Materials

Fura-2 was from Invitrogen (cat. no. F1221, Eugene, OR, USA). Coverslips Menzel-Gläser Ø25 mm #1 were from Thermo Scientific (Darmstadt, Germany). All salts and other reagents were purchased from PanEco (Moscow, Russia).

Cardiotoxins were isolated from cobra venoms as described [6]. The structures of isolated CaTxs were confirmed by mass spectrometry and purity by analytical reversed phase HPLC.

### 4.2. Cardiomyocyte Preparation

Hearts were dissected from anesthetized animals (pentobarbital, 50 mg/kg i.p.), and solutions for retrograde perfusion and ventricular myocytes isolation were prepared based on a “low-Ca^2+^ medium” containing (in mM): NaCl, 80; KCl, 10; KH_2_PO_4_, 1.2; MgSO_4_, 5; glucose, 20; taurine, 50; L-arginine, 1; and HEPES, 10 (pH 7.2), as described previously [42]. Isolated cardiomyocytes were stored in low-Ca^2+^ medium supplemented with 200 μM CaCl_2_. Only rod-shaped cardiomyocytes with clear striations were used. Cells were stored at 4 °C in low calcium Hanks’ balanced salt solution (HBSS) with the following composition (in mM): 138 NaCl, 1.3 CaCl_2_, 0.4 MgSO_4_, 0.5 MgCl_2_, 5.3 KCl, 0.45 KH_2_PO_4_, 4 NaHCO_3_, 0.3 Na_2_HPO_4_, 10 glucose, and 20 HEPES (pH 7.36 at 29–30 °C was adjusted with NaOH). The cells were used for study within 6–7 h after isolation.

### 4.3. Dye Loading

For staining with a fluorescent calcium-sensitive ratiometric probe Fura-2, the cells were placed in the center of a round cover slip with a diameter of 25 mm and a thickness of 0.17 mm in a drop of 200 μL, then the cells were allowed to precipitate for 2 min. Using a pipette, the medium was replaced with 200 µL of HBSS containing 5 µM Fura-2AM. Cells were incubated for 40 min at 30 °C in the dark. Then, the medium was replaced with HBSS without dye with a pipette, a coverslip was mounted in a special measuring cell for an inverted microscope, and the cells were washed again. The volume of the medium was adjusted to 1 mL and the measuring cell was placed on the microscope stage for the experiment.

### 4.4. Fluorescence Microscopy

Fura-2 fluorescence in cardiomyocytes was measured using a Cell Observer fluorescent station based on an AxioVert 200 M motorized inverted microscope (Carl Zeiss AG, Oberkochen, Germany) equipped with a 10× PlanApochromat objective, an Orca-Flash R2 monochrome camera (Hamamatsu Photonics K.K., Iwata City, Japan), and a system for a high-speed change in excitatory filters Ludl MAC 5000 (Ludl Electronic Products, Hawthorne, NY, USA). Fura-2 fluorescence was excited with an HBO100 mercury lamp in two channels using a set of 21HE light filters (Carl Zeiss AG, Oberkochen, Germany): excitation 1—340 ± 15 nm (channel 340); excitation 2—387 ± 8 nm (channel 380); FT 409 beam splitter; and 510 ± 45 nm emission filter. The exposure time for each of the channels was 100 ms. The registration frequency was 1 frame per 5 s. The power of the light source was set to the minimum value that provided an acceptable signal-to-noise ratio. In control experiments with the selected recording parameters, no effect of illumination on the Fura-2 signal was detected for 30 min or more.

### 4.5. Reagents Application

To ensure uniform distribution of the added CaTx in the cell incubation medium, the substances were applied as follows: from 1 mL of HBSS in the measuring cell, half was taken, and a solution of CaTx with double concentration was prepared in a separate microtube. After that, the CaTx solution was returned to the measuring cell and gently mixed twice with a micropipette. In the control experiments, no noticeable effect of the taking and mixing of the medium on the Fura-2 signal was observed.

### 4.6. Image Processing

The two-channel series of images was processed in Image J/FiJi (NIH, Bethesda, MD, USA). After determining and subtracting the background signal, the images were smoothed with a simple anti-aliasing filter with a 3 × 3 px grid and the ratio of the data for the 340 nm channel to those of the 380 nm channel, in 32-bit format, was calculated. Next, ROIs (regions of interest) corresponding to the position of the cardiomyocyte were selected in order to capture only the area of the cell, regardless of its movement during the contraction. A table of the ratio of Fura-2 fluorescence signals at 340 and 380 nm excitation versus time was calculated. In some cases, to determine the moment of destruction of the integrity of the plasmalemma by the outflow of the fluorescent probe, the channel for registering the calcium-bound form of the dye (channel 340) was used. Origin 2016 (OriginLab Corporation, Northampton, MA, USA) and GraphPad Prism 8 (GraphPad Software Inc., Boston, MA, USA) were used to plot the graphs. 

### 4.7. Data Analysis and Statistics

Paired *t*-test and one-way ANOVA with Tukey’s post hoc test were used for the comparison of two or multiple groups, respectively. The difference was considered statistically significant at *p* < 0.05. All data are presented as mean ± standard error (S.E.)

## 5. Conclusions

Thus, a comparison of the effects of CaTxs of different types and groups on rat cardiomyocytes showed that the P-type toxins produced stronger effects than the S-type ones, and that group II toxins were more active than those of group I. All toxins exerted close impacts on the cardiomyocyte calcium overload and contracture. However, the effects strongly depended on the structures of the loops I and II. We suggest that CaTxs of the P-type (with Pro30 in the loop II) from group II (with one Pro9 in loop I) possess the highest toxicity. The observed variations may be explained by the differences in the membrane-damaging activity of CaTxs.

## Figures and Tables

**Table 1:** Effects of CaTxs at different concentrations on cardiomyocytes.

Toxin	Concentration	Parameter		
		Cells with Hypercontracture, %	Time to Hypercontracture, s	Time to Peak of Ca^2+^ Concentration, s
CT2No	10 μg/mL	100	393 ± 207	396 ± 217
	25 μg/mL	100	162 ± 86	152 ± 87
CT1Nk	10 μg/mL	100	503 ± 307	520 ± 318
	25 μg/mL	100	312 ± 148	299 ± 137
CT1No	10 μg/mL	24 ± 21	933 ± 707	909 ± 634
	25 μg/mL	89 ± 13	736 ± 397	747 ± 387
CT2Nh	25 μg/mL	18 ± 17	1328 ± 577	1255 ± 615
	100 µg/mL	100	531 ± 148	518 ± 163
CT1Nh	25 μg/mL	7 ± 12	1522 ± 177	1420 ± 223
	100 µg/mL	74 ± 29	1132 ± 342	1151 ± 295

## Data Availability

All data obtained in this study are contained within the article.
